# Interest and Inflation Risk: Investor Behavior

**DOI:** 10.3389/fpsyg.2016.00390

**Published:** 2016-03-18

**Authors:** María de la O González, Francisco Jareño, Frank S. Skinner

**Affiliations:** ^1^Department of Economic Analysis and Finance, University of Castilla-La ManchaAlbacete, Spain; ^2^Department of Economics and Finance, Brunel UniversityUxbridge, UK

**Keywords:** unexpected inflation, interest rates, stock return, business cycle, investor behavior

## Abstract

We examine investor behavior under interest and inflation risk in different scenarios. To that end, we analyze the relation between stock returns and unexpected changes in nominal and real interest rates and inflation for the US stock market. This relation is examined in detail by breaking the results down from the US stock market level to sector, sub-sector, and to individual industries as the ability of different industries to absorb unexpected changes in interest rates and inflation can vary by industry and by contraction and expansion sub-periods. While most significant relations are conventionally negative, some are consistently positive. This suggests some relevant implications on investor behavior. Thus, investments in industries with this positive relation can form a safe haven from unexpected changes in real and nominal interest rates. Gold has an insignificant beta during recessionary conditions hinting that Gold can be a safe haven during recessions. However, Gold also has a consistent negative relation to unexpected changes in inflation thereby damaging the claim that Gold is a hedge against inflation.

## Introduction

A lot of previous financial research assumes that investors are rational agents, so they try to optimize wealth and minimize risk (Campbell, 2006). Thus, the study of two relevant sources of risk such as interest and inflation rate movements is very interesting for deepening on the analysis of investor behavior as well as for portfolio managers. Furthermore, the recent financial crisis confirms that investor behavior changes over time (Ferrando et al., 2015), so this analysis is really challenging to achieve a better understanding of investor behavior. Moreover, according to Blackburn et al. (2014), investor behavior may depend on different factors that affect the investment or trading decision. Therefore, aspects such as the sector that traded stock belongs to and the business cycle—among others—apparently impact on investment behavior.

The US stock market is a world reference market so unexpected changes in US nominal interest rates can affect stock markets worldwide. Moreover, being the most active equity market with the longest series of detailed quality data, the US stock market is a natural laboratory to study the relationships between unanticipated inflation and its co-dependents, unanticipated changes in real, and nominal interest rates, in detail by sector and by varying economic conditions. It is important to examine these relations by sector because there is no reason to expect that individual sector returns are always inversely related to unanticipated changes in inflation and real and nominal interest rates. For instance, according to the flow through model of Estep and Hanson (1980), the impact of inflation on stock prices can be neutral if the firm can pass on inflationary price increases to consumers. If so, then an investment in stocks can serve as a safe haven for investors as stock prices rise with inflation. Additionally, the impact of unanticipated real and nominal interest rate changes can vary by sector depending upon the characteristic leverage and competitive structure of the sector. Moreover, it is also important to examine these relations by time period as conventionally inverse relations can turn positive as economic conditions change. For instance, it could be that an investment in cyclical industries such as the Industrial sector can have a positive relation with unanticipated inflation during boom economics conditions that turns negative during recessions.

**Figure 1:**
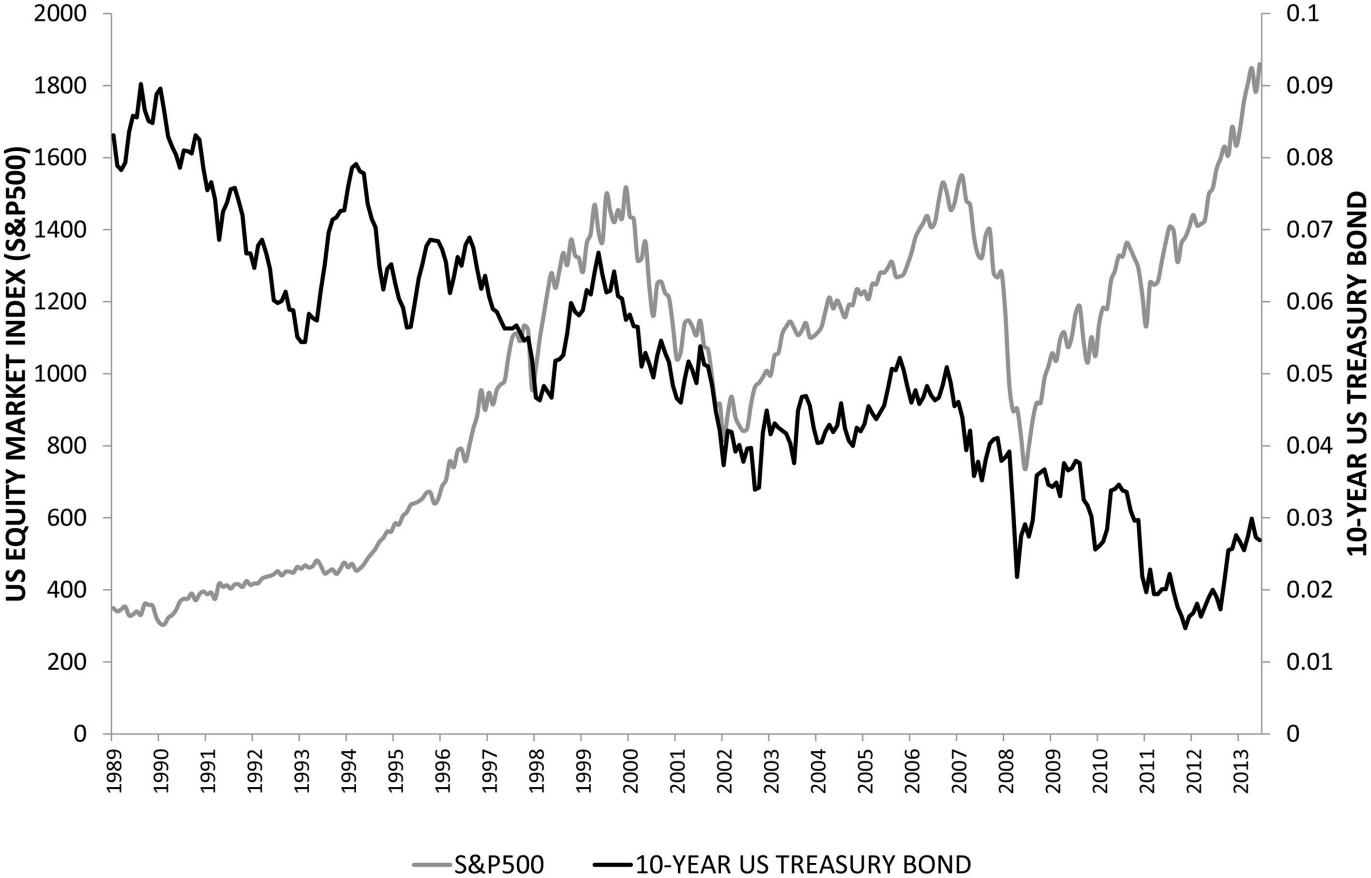
**Evolution of the US equity market index (S&P 500) and the 10-year US Treasury Bond yield from September 1989 to February 2014**.

Figure 1 presents the evolution of the US stock market index (S&P500) and the 10-year Treasury bond yield from September 1989 to February 2014. On the one hand, the US stock market exhibits an increasing trend during most of the period, only interrupted by the dot-com bubble burst in 2000 and the global financial crisis at the end of 2007 (Bartram and Bodnar, 2009). On the other hand, the 10-year Treasury bond yield shows a decreasing tendency. So at first glance, we observe clear evidence of the inverse association between US stock market returns and changes in the nominal interest rate. However, we raise the question of whether this inverse relation is consistent by sub-period and whether this inverse association is maintained when we break down unexpected changes in the nominal interest rate into unexpected changes in the real interest and inflation rates, especially when we examine these relations by sector, industry, and by economic condition. Thus, the crucial aim of this paper is to analyze the details of the relation between returns on US stocks and unexpected changes in nominal and real interest rates and inflation, because the investor behavior may be quite different depending on the sector, industry, and the state of the economy.

The literature examines the sensitivity of stock returns to unexpected changes in nominal interest rates finding a negative and significant relationship between stock returns and unanticipated changes in nominal interest rates. See O'Neal (1998), Fraser et al. (2002), Hevert et al. (1998a,b), Tessaromatis (2003), Jareño (2006, 2008), Ferrer et al. (2010), Korkeamäki (2011), Ferrando et al. (2015), and Campos et al. (2016) as examples. Some have examined these relations for the overall stock market (Elyasiani and Mansur, 1998; Oertmann et al., 2000; Shamsuddin, 2014) while others have mainly studied these relations for financial companies (Flannery and James, 1984; Fraser et al., 2002; Staikouras, 2003, 2006; Au Yong and Faff, 2008; Drehmann et al., 2010; Ballester et al., 2011; Memmel, 2011; Bessler and Kurmann, 2012; Abdymomunov and Gerlach, 2014) or for Utilities (Sweeney and Warga, 1986). Others have deepened the analysis by decomposing unexpected changes in nominal interest rates into unexpected changes in real interest and unexpected inflation rates (Tessaromatis, 2003; Jareño, 2006, 2008; Jareño et al., 2016).

This paper is one of the few to estimate the stock return response to unexpected shocks in the nominal interest rate and its components, unexpected changes in the inflation rate, and the residual that we interpret as unexpected changes in the real interest rate. Moreover, this study tries to approximate investor behavior analysing sector stock response to changes in sources of risk in different scenarios. To accomplish this task, we use an extension of the Stone (1974) two-factor model proposed in Jareño (2006) and, partly, in Jareño (2008), and Jareño and Navarro (2010). Using this approach, we make two contributions. First, we analyze these relations at the sector, sub-sector and industry level. Thus, we estimate not only the relation between stock returns and unexpected nominal interest rate changes but also the relations between stock returns and unexpected changes in the real interest and inflation rates by sector, sub-sector, and individual industries. Second, we examine a long time period, from September 1989 to February 2014. This period encompasses a wide variety of economic conditions, including one of the longest expansion periods for the US economy, one of the most severe credit contractions in living memory and several recessions. This sample variation in economic conditions allows us to explore the stability of these relations overall, and by sector, sub-sector, and industry. This detailed investigation into the stability of these relations allows us to search for special industries whose response to unexpected changes in nominal and real interest rates, and unanticipated inflation, is consistent, either positive or negative, thereby providing valuable information for investors and policy makers who have to consider these important sources of systematic risk.

In general, we find that investor behavior seems to be quite different over time (according to the business cycle) and by sector. Specifically, some financial (as well as non-financial) sectors have insignificant relations and we even find some contrary results when examining the relations by sector, sub-sector, and industry. Some industries have a consistent significant positive relation between stock returns and unexpected changes in real and nominal interest rates. Interestingly, Gold, among others, has a negative relation to unanticipated inflation in the overall sample and in the contraction and expansion sub-periods suggesting that it is exposed to inflation risk.

The rest of the paper is structured as follows. Section Materials and Methods present the main methodology used in this research. Section Data describes the data and variables included in our empirical analysis. Section Empirical Results comments on the results of our research, and finally, Section Discussion makes concluding remarks.

## Materials and methods

In this section, we explain how we measure unexpected changes in the nominal rate of interest. Then we explain how we measure the expected rate of inflation that is used as an input to decompose the unexpected change in nominal interest rates into unexpected changes in inflation and unexpected changes in the real rate of interest. Finally, we describe how we classify the state of the economy into expansion and non-expansion (contraction) states.

### Unexpected changes in nominal interest rates

Sweeney and Warga (1986), Kane and Unal (1988), Bartram (2002), Oertmann et al. (2000), and Olugbode et al. (2014) amongst others use changes in long-term interest rates as a proxy for unexpected changes in nominal interest rates because long term interest rates incorporate the expectations of economic agents and because long term interest rates are important as they determine the cost of corporate borrowing. Thus, long term interest rates strongly influence the investment decisions of firms and therefore affect the value of companies. Alternative proxies for unexpected changes in nominal interest rates such as forecast error of an empirical ARIMA process for long term interest rates or survey data on the US federal funds rate (Benink and Wolff, 2000) have their own advantages and disadvantages (Froot, 1989) so no one proxy dominates. Therefore, we follow common practice and use the first difference of the long-term interest rate as a proxy for unexpected changes in the nominal interest rate.

The returns on Treasury securities for different maturities are usually used as risk-free interest rate proxies because Treasury securities are commonly assumed to have no default risk. Of the long term maturities, 10 years tends to be the most liquid and accurately estimated as the Fed has continuously auctioned 10 year Treasury notes throughout our sample period so there is always a recently issued 10-year note that the Fed can use to accurately estimate 10 year treasury yields. Therefore, we use changes in the 10-year US Treasury bond yields, as reported by the Federal Reserve Bank of New York, as our approximation for unexpected changes in the nominal interest rate. We repeat our empirical results using 3-month, 1-, 3-, and 5- year US Treasury bond yield changes and find the results are very similar. These are available from the corresponding author upon request.

### Expected inflation rates

Although, previous studies have applied a variety of methodologies to estimate expected inflation rates, a lot of related, and crucial papers (Schwert, 1981; Pearce and Roley, 1988; Fraser et al., 2002; Mestel and Gurgul, 2003; Jareño, 2008), use simple time series ARIMA models to estimate the expected inflation component. These studies assume that the current total inflation rate (π_t_) can be broken down into the sum of its expected ($\pi_{\text{t}}^{\text{e}}$) and unexpected ($\pi_{\text{t}}^{\text{u}}$) components. Thus, the expected component is estimated using ARIMA models thereby assuming that this component depends upon its own past series. Then the forecast errors from the ARIMA model form our estimate of unanticipated changes in inflation. We also use ARIMA models because authors, such as Joyce and Read (2002) and Browne and Doran (2005), observe similar results using ARIMA and other alternative and more sophisticated procedures. These models, in contrast to structural models, do not need additional information for doing forecasts, because they use lagged inflation values. We have repeated this procedure until the end of sample, with one-step-ahead forecast, obtaining the expected component of inflation rate.

We use the Akaike information criterion (AIC) to choose the ARMA (1, 0) process to predict the month-to-month annualized inflation rate. Therefore, we suppose short-sighted expectations as in Leiser and Drori (2005). Unit root tests confirm that inflation rate is a I(1) series, so this result is consistent with short-sightedness expectations. That is,

$$E_{t}\left(\pi_{t,\;t\;+\;1}\right)=\rho \pi_{t\;-\;1,\;t}$$

In other words, expectations are formed in part [ρ according to the ARMA(1, 0) process], as of time t for the expected rate of annualized inflation π over the next month *t*+1 based on the most recent monthly annualized inflation rate that evolved from *t*−1 to *t*.

### Unexpected changes in the real rate of interest

As mentioned above, we use changes in the 10-year US Treasury bond yield as our approximation for unexpected changes in the nominal interest rate. To obtain unexpected changes in the real rate of interest we assume the Fisher approximation and subtract the expected rate of inflation *E*_*t*_(π_*t, t*+1_) as estimated above from the nominal rate of interest *i*_*t*_.

$$\begin{array}{l} r_{t}\approx i_{t}-E_{t}\left(\pi_{t,t\;+\;1}\right) \end{array}$$

Then, changes in the above relation form our approximation for unexpected changes in in the real rate of interest.

### The Stone (1974) two-factor model

The literature focuses mainly on the Stone (1974) two-factor model to measure the interest rate sensitivity of stock returns (Lynge and Zumwalt, 1980; Sweeney and Warga, 1986; O'Neal, 1998; Bartram, 2002; Fraser et al., 2002; Soto et al., 2005; Staikouras, 2005; Jareño, 2006, 2008; Ferrer et al., 2010). We use an extension of the Stone (1974) model that decomposes unexpected changes in the nominal interest rate into unexpected changes in real interest and inflation components in the nature of Tessaromatis (2003), Cornell (2000), Jareño (2006, 2008). However, all of these studies do not examine any sector other than the financial or the utility sector. Thus, we propose an analysis at the sector, sub-sector and industry level using an extension of the Stone (1974) model.

Typically, studies of interest rate sensitivity of stock returns start from the Capital Asset Pricing Model CAPM augmented by unexpected changes in nominal interest rates (Stone, 1974) to better explain the stochastic process that generates security returns. Therefore, adjusting Arango et al.'s (2002) model of stock returns by sector, sub-sector and industry we have,

$$r_{jt}=\alpha_{j}+\beta_{j}\cdot r_{mt}+\gamma_{j}\cdot \Delta i_{t}^{\;u}+\varepsilon_{jt}$$

where *r*_*jt*_ is the stock (sector, sub-sector or industry) *j* return in month *t*, β_*j*_ shows the stock sensitivity to market movements, *r*_*mt*_ is the return on the market portfolio, ${\Delta i}_{t}^{u}$ represents unexpected changes in nominal interest rates, and, finally, ε_*jt*_ is the error term.

We extend the Stone (1974) model by applying the Fisher approximation to break down nominal interest rates *i*_*t*_ into real interest *r*_*t*_ and expected inflation *E*_*t*_(π_*t, t*+1_) components. Taking the first difference in interest rates as unexpected changes in nominal interest rates at time t, we then have unexpected changes in the nominal interest rate ${\Delta i}_{t}^{u}$ as a linear combination of unexpected changes in the real rate Δ*r*_*t*_ and unexpected changes in the anticipated inflation rate Δ*E*_*t*_(π_*t, t*+1_). Thus, the second model estimated in this paper is the following:

$$r_{jt}=\alpha_{j}+\beta_{j}\cdot r_{mt}+\beta_{jr}\cdot \Delta r_{t}+\beta_{j\pi }\cdot \Delta E_{t}^{ORT}\left(\pi_{t,t+1}\right)+\varepsilon_{jt}$$

where *r*_*jt*_ is the stock (sector, sub-sector or industry) *j* return in month *t*, β_*j*_ shows the stock sensitivity to market movements, *r*_*mt*_ is the return on the market portfolio, Δ*r*_*t*_ represents unexpected changes in real interest rates, $\Delta E_{t}^{ORT}\left(\pi_{t,t+1}\right)$ shows shocks in the expected inflation rate (hereafter, unexpected changes in the inflation rate that we later explain is orthogonalized), and finally, ε_*jt*_ is the error term.

To avoid possible high collinearity between the explanatory variables, the financial economics literature uses some orthogonalization procedure. In Table 1A we observe a high, significant correlation between unexpected changes in real interest and unexpected changes in the inflation rate (about −83%). We also find two other significant correlations that we do not need to orthogonalize as they do not simultaneously occur in our model; the first is between changes in real and nominal interest rates (about 44%) and the second is between unexpected changes in inflation and nominal interest rates (about 15%). So, as in Lynge and Zumwalt (1980), Flannery and James (1984), Sweeney (1998), and Fraser et al. (2002), we orthogonalize the relation between unexpected changes in the real interest rate and unexpected changes in the inflation rate by regressing changes in the unexpected inflation rate on a constant and changes in the unexpected real interest rate using ordinary least squares regression. The residual from this regression forms our proxy for the orthogonalized unexpected change in the inflation rate. Thus, the effect of each factor is isolated and the movement that remains is captured by the residuals.

**Table 1:** **Correlation matrix between explanatory variables included in the model**.

	***r_*mt*_***	***${\Delta i}_{t}^{u}$***	***Δr_*t*_***	***ΔE*_*t*_(π_*t, t*+1_)**
**(A) BEFORE ORTHOGONALIZATION PROCEDURE**				
*r_*mt*_*	1.000			
*${\Delta i}_{t}^{u}$*	0.067	1.000		
*Δr_*t*_*	0.071	0.436^***^	1.000	
Δ*E*_*t*_(π_*t, t*+1_)	−0.036	0.145^**^	−0.827^***^	1.000
	***r**_*mt*_*	*${\Delta i}_{t}^{u}$*	*Δ**r**_*t*_*	$\Delta E_{t}^{ORT}\left(\pi_{t,t+1}\right)$
**(B) AFTER ORTHOGONALIZATION PROCEDURE**				
*r_*mt*_*	1.000			
*${\Delta i}_{t}^{u}$*	0.067	1.000		
*Δr_*t*_*	0.071	0.436^***^	1.000	
$\Delta E_{t}^{ORT}\left(\pi_{t,t+1}\right)$	0.040	0.900^**^	−0.000	1.000

*r_mt_ is the return on the market portfolio; ${\Delta i}_{t}^{u}$ represents (unexpected) changes in nominal interest rates (10-year US Treasury bond yield changes); Δr_t_ changes in real interest rates and $\Delta E_{t}^{ORT}\left(\pi_{t,t+\text{1}}\right)$ shows unexpected changes in the orthogonalized inflation rate*.

*, **, *** *indicate statistical significance at the 10, 5, and 1% levels, respectively*.

We choose this orthogonalization method because this is in line with the aim of this research, which is to estimate the response of stock (sector, sub-sector and industry) returns to unanticipated changes in nominal interest rates and its' decomposition, unexpected changes in real, and unexpected changes in inflation rates. Therefore, we analyze direct and indirect effects of interest rate shocks and obtain clear economic intuition. We find similar results to those obtained without orthogonalizing and also very similar results when we interchange the dependent and independent variables. Thus, our results seem to be robust, since this orthogonalization process evidently only eliminates the correlation between variables.

The final correlations between explanatory variables included in our model are reported in Table 1B. Notice that the correlation between unexpected changes in the real interest rate and unexpected changes in the inflation rate is zero.

### State of the economy

Like Veronesi (1999), Knif et al. (2008), and Díaz and Jareño (2009, 2013), we assume that the stock market response to unanticipated changes in nominal and real interest and inflation rates depends on the business cycle. Therefore, we need to classify the state of the economy. We follow the National Bureau of Economic Research (NBER's) classification, but this is only available until June 2009. Therefore, we extend this classification by examining the evolution of the annual growth of the US GDP after seasonal adjustment (as in Díaz and Jareño, 2013) in order to identify expansion and non-expansion (contraction) months. Specifically, a contraction begins with a recession as defined as two or more quarters of negative seasonally adjusted growth. A contraction continues throughout the recovery period and converts to an expansion only when seasonally adjusted GDP rises above the peak of GDP just prior to the recession.

**Figure 2:**
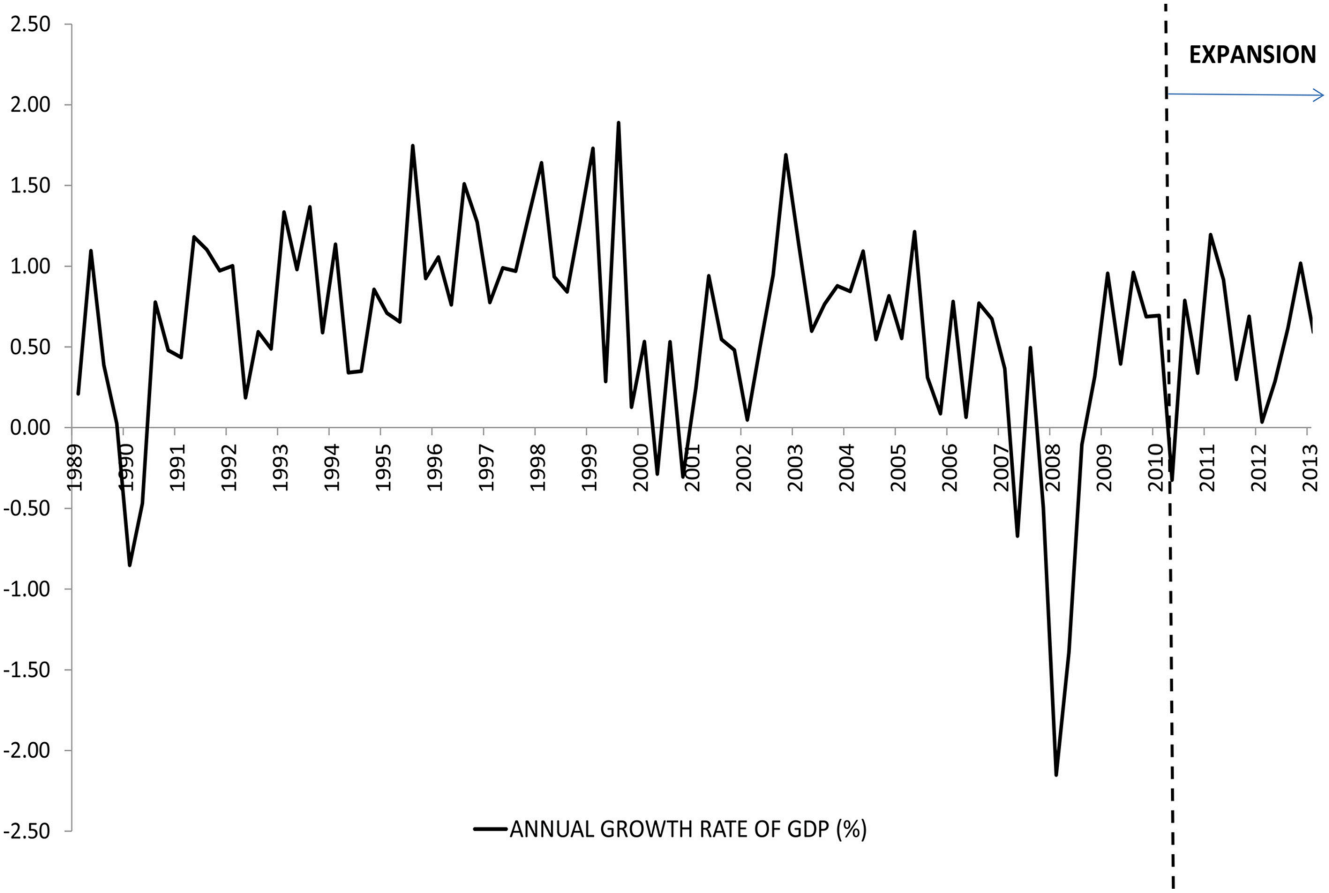
**Evolution of the annual growth rate of GDP (%) after seasonal adjustment**.

Table 2 and Figure 2 show the business cycle timing. This classification follows NBER announcements for the most part and divides the state of the economy in expansion and contraction months. During the 292 month period, from November 1989 to February 2014, the US Economy was in an expansion during 237 months and in contraction during 55 months. So, there were three contraction and four expansion periods.

**Table 2:** **US business cycle expansions and contractions**.

**Period**	**State of the economy—Number of months**
November 1989–June 1990	Expansion—8 months
July 1990–February 1991	Contraction—8 months
March 1991–March 2001	Expansion—121 months
April 2001–November 2001	Contraction—8 months
December 2001–December 2007	Expansion—73 months
January 2008–March 2011	Contraction—39 months
April 2011–February 2014	Expansion—35 months
Total expansion months	237 months
Total contraction months	55 months
TOTAL	292 months

*Source: NBER (The National Bureau of Economic Research)*.

*NBER's classification is only available until June 2009, so we extend it by classifying all months with growth above the previous peak as expansion and all other months as contraction according to the US GDP after seasonal adjustment (Díaz and Jareño, 2013). For more information, please see the guide to the National Income & Products Accounts of the United States (NIPA) www.bea.gov/national/pdf/nipaguidepdf*.

## Data

Our data set includes monthly indices for the US sector, sub-sector, and industries from November 1989 to February 2014, 292 monthly observations in all. The US sector index is based on the “Global Industry Classification Standard” GICS as developed by Morgan Stanley Capital International and Standard &Poor's. This classification aims to enhance the investment research and asset management process for financial professionals worldwide. Also, GICS is the result of numerous discussions with asset owners, portfolio managers and investment analysts. Finally, this classification is designed to respond to the global financial community's need for an accurate, complete, and standard industry definition. The sub-sector and individual industry indices are refinements of the GICS compiled by and obtained from Bloomberg. We also use the monthly S&P500 market index from Bloomberg and the monthly 10-year US Treasury yields from the Federal Reserve. Finally, we use the monthly expected inflation rates as explained in Section Expected Inflation Rates.

The Supplementary Material Table A reports the sector, sub-sector, and industry classifications according to the GICS combined with the Bloomberg refinements. In this paper we analyze 10 sectors, subdivided into 33 sub-sectors, and further refined into 82 industries. The largest US industry sectors by market capitalization (as of April 29, 2010), are Information Technology (19.02%), and Financials (16.58%). There are five other noteworthy sectors with weights around 10%, Consumer Discretionary, Consumer Staples, Energy, Health Care, and Industrials.

Table 3 reports the monthly returns for the S&P500 Index and the US sector indices. The mean and median returns for all sectors and the market are positive and fairly large; the mean monthly return is 58 basis points or 7.2% on an annual basis. Changes in the 10-year US bond yield, our proxy for the unexpected changes in the nominal interest rate, are negative as are unexpected changes in real interest and inflation rates, reflecting the decreasing trend of long-term interest rates as shown earlier in Figure 1. The most volatile sector is Information Technology, followed by Financials. Also, sector and market stock return volatilities are higher than nominal and real interest and inflation rate volatilities. Except for the real rate of interest, all the variables exhibit negative skewness and all variables have excess kurtosis, especially for unexpected changes in the inflation rate. The Jarque-Bera test rejects the null hypothesis of Normal distribution in all cases at the 5% significance level except for unexpected changes in nominal interest rates.

**Table 3:** **Descriptive statistics of sector and market returns, 10-year US Treasury bond yield changes (nominal interest rates), and real interest and expected inflation rate changes**.

**Sector returns and risk factors**	**Mean**	**Median**	**Max**.	**Min**.	**Std. Dev**.	**Skew**.	**Kurtosis**	**JB statistic**	**ADF statistic**	**PP statistic**	**KPSS statistic**
S1 Consumer discretionary	0.008	0.012	0.171	−0.213	0.052	−0.501	4.402	36.135^***^	−15.144^***^	−15.076^***^	0.092
S2 Consumer staples	0.009	0.011	0.144	−0.126	0.038	−0.363	4.631	38.798^***^	−15.839^***^	−15.842^***^	0.148
S3 Energy	0.009	0.009	0.171	−0.198	0.053	−0.333	4.262	24.779^***^	−17.940^***^	−17.931^***^	0.041
S4 Financials	0.006	0.014	0.202	−0.305	0.065	−0.941	6.550	196.457^***^	−15.003^***^	−15.042^***^	0.223
S5 Health care	0.009	0.013	0.151	−0.133	0.045	−0.320	3.383	6.778^**^	−17.331^***^	−17.348^***^	0.214
S6 Industrials	0.008	0.014	0.164	−0.209	0.051	−0.724	5.023	75.313^***^	−15.672^***^	−15.671^***^	0.089
S7 Information technology	0.008	0.014	0.201	−0.328	0.075	−0.625	4.667	52.825^***^	−16.972^***^	−16.980^***^	0.140
S8 Materials	0.007	0.011	0.216	−0.249	0.058	−0.405	4.841	49.182^***^	−16.930^***^	−16.938^***^	0.032
S9 Telecommunications	0.005	0.012	0.283	−0.168	0.056	−0.128	5.134	56.201^***^	−17.104^***^	−17.167^***^	0.109
S10 Utilities	0.006	0.011	0.128	−0.151	0.044	−0.655	4.156	37.146^***^	−15.461^***^	−15.558^***^	0.051
Market portfolio return	0.006	0.011	0.106	−0.186	0.043	−0.804	4.623	63.511^***^	−15.940^***^	−16.002^***^	0.142
10-year US bond yield changes	−1.8E-04	−2.0E-04	0.008	−0.009	0.003	−0.089	3.621	5.070^*^	−15.298^***^	−15.211^***^	0.019
Real interest rate changes	−8.0E-05	−2.0E-05	0.021	−0.017	0.005	0.285	5.484	79.037^***^	−8.201^***^	−12.449^***^	0.018
Expected inflation rate changes	−9.8E-05	−7.5E-05	0.021	−0.026	0.004	−0.657	11.645	930.394^***^	−10.212^***^	−10.523^***^	0.020

*This table presents the main descriptive statistics of monthly sector and market portfolio returns and changes in 10-year US Treasury bond yields over the period from November 1989 to February 2014. They include mean, median, maximum (Max.), and minimum (Min.) values, standard deviation (Std. Dev.), and Skewness (Skew.) and Kurtosis measures. JB denotes the statistic of the Jarque-Bera test for normality. The results of the augmented Dickey-Fuller (ADF) and Phillips-Perron (PP) unit root tests and the Kwiatkowsky-Phillips-Schmidt-Shin (KPSS) stationarity test are also reported in the last three columns. As usual*,

*, **, *** *indicate statistical significance at the 10, 5, and 1% levels, respectively*.

We examine the stationary of the variables in the second part of Table 3 using the augmented Dickey-Fuller (ADF) and Phillips-Perron (PP) unit root tests and the Kwiatkowski-Phillips-Schmidt-Shin (KPSS) stationary test. Similar to Jareño (2008), Czaja et al. (2009), and Ballester et al. (2011) these tests corroborate that the series of sector and stock market portfolio returns, real interest rate, and expected inflation rates, are stationary.

In order to obtain continuously compounded returns for industry sectors, sub-sectors, and industries *r*_jt_, we compute the log relatives using the closing index of the last day of the current month *P*_*jt*_ relative to the closing index of last day of the previous month *P*_*jt*−1_. That is,

$$r_{jt}=\log\left(\frac{P_{jt}}{P_{jt\;-\;1}}\right)$$

To avoid income smoothing, we use index values net of dividends. We use the S&P500 index as a suitable representative of the US stock market and compute the log relative return in an analogous way as in (3) to obtain market log-returns.

## Empirical results

We estimate two models, (1) examines the relation between stock returns and unanticipated changes in nominal interest rates and (2) estimates the relation between stock returns and unanticipated changes in real interest and inflation rates. Both models are applied separately by sector, sub-sector, and industry and are estimated throughout the sample period and during expansion and contraction economic sub-periods from September 1989 to February 2014. We estimate models (1) and (2) separately using the “seemingly unrelated regression” SUR technique (Zellner, 1962) for each of the sector, sub-sector, and industry samples, six SUR regressions in all, thereby taking into account possible contemporaneous correlation in the error terms across sectors, sub sectors, and industries as well as heteroskedasticity.

### Results at the sector level

We regress models (1) and (2) at the sector level and we report the results in Table 4. Table 4A reports the results for the entire sample period and Tables 4B,C report the results for the contraction and expansion sub-periods, respectively. The adjusted R squares of both models are very similar where for model 1, the adjusted R square ranges between about 65% for Information Technology, and about 24% for Utilities. All sectors exhibit a positive and significant market beta for both models overall and in the contraction and expansion sub-periods. While the betas are different in the contraction and expansion sub-periods, there is no discernible pattern to these differences. The beta coefficients are nearly the same by sector for the two models. For the overall period, the beta coefficients vary between the least risky Utilities 0.47 to the most risky Information Technology sector 1.38.

**Table 4:** **Coefficients of sector stock returns to variations in nominal interest rates (Model 1) and real interest and expected inflation rates (Model 2)**.

***Sector***	**Model 1**			**Model 2**			
	***r_*mt*_***	***${\Delta i}_{t}^{u}$***	***Ad. R^2^***	***r_*mt*_***	***Δr_*t*_***	**$\Delta E_{t}^{ORT}\left(\pi_{t,t+1}\right)$**	***Ad. R^2^***
**(A) TOTAL SAMPLE (FROM NOV. 1989 TO FEB. 2014)**							
S1 Consumer discretionary	1.075^***^	0.517	0.795	1.074^***^	0.452	0.212	0.795
S2 Consumer staples	0.593^***^	−2.221^***^	0.445	0.594^***^	−0.704^*^	−2.076^***^	0.443
S3 Energy	0.769^***^	1.538^*^	0.406	0.765^***^	1.109^**^	0.852	0.409
S4 Financials	1.278^***^	−0.604	0.708	1.279^***^	−0.338	−0.427	0.708
S5 Health care	0.712^***^	−1.706^**^	0.453	0.713^***^	−0.496	−1.637^*^	0.451
S6 Industrials	1.072^***^	0.394	0.820	1.072^***^	0.009	0.477	0.820
S7 Information technology	1.385^***^	2.284^**^	0.648	1.384^***^	0.641	2.213^**^	0.647
S8 Materials	1.061^***^	1.513^*^	0.628	1.059^***^	0.754^*^	1.157	0.628
S9 Telecommunications	0.844^***^	−1.552	0.419	0.845^***^	−0.527	−1.418	0.417
S10 Utilities	0.467^***^	−3.495^***^	0.238	0.469^***^	−1.308^***^	−3.078^***^	0.238
**(B) CONTRACTION SUB-PERIOD**							
S1 Consumer discretionary	1.232^***^	−2.453^**^	0.880	1.232^***^	−0.699	−2.414^**^	0.878
S2 Consumer staples	0.566^***^	−2.306^**^	0.687	0.568^***^	−1.050^**^	−2.107^**^	0.688
S3 Energy	0.781^***^	4.298^**^	0.536	0.778^***^	1.814^***^	3.986^*^	0.533
S4 Financials	1.496^***^	−3.218	0.753	1.500^***^	−1.760	−2.816	0.752
S5 Health care	0.672^***^	−1.412	0.637	0.673^***^	−0.607	−1.305	0.631
S6 Industrials	1.290^***^	−1.860^**^	0.927	1.291^***^	−0.695	−1.763^*^	0.926
S7 Information technology	1.239^***^	3.231^*^	0.767	1.235^***^	1.670^*^	2.869	0.768
S8 Materials	1.234^***^	1.170	0.857	1.232^***^	0.674	1.010	0.855
S9 Telecommunications	0.602^***^	−3.151	0.346	0.603^***^	−1.202	−2.975	0.336
S10 Utilities	0.571^***^	−0.873	0.439	0.573^***^	−0.503	−0.753	0.430
**(C) EXPANSION SUB-PERIOD**							
S1 Consumer discretionary	1.001^***^	1.458^**^	0.755	0.997^***^	0.796^**^	0.953	0.756
S2 Consumer staples	0.611^***^	−2.163^***^	0.378	0.611^***^	−0.505	−2.199^**^	0.376
S3 Energy	0.748^***^	0.534	0.343	0.739^***^	1.152^*^	−0.6514	0.349
S4 Financials	1.152^***^	0.118	0.685	1.150^***^	0.290	−0.185	0.684
S5 Health care	0.735^***^	−1.777^*^	0.394	0.735^***^	−0.386	−1.839^*^	0.391
S6 Industrials	0.953^***^	1.013^*^	0.763	0.954^***^	0.164	1.113^*^	0.762
S7 Information technology	1.473^***^	2.087^*^	0.610	1.477^***^	0.034	2.648^*^	0.609
S8 Materials	0.969^***^	1.508	0.510	0.965^***^	0.789	1.026	0.509
S9 Telecommunications	0.989^***^	−0.787	0.460	0.990^***^	−0.367	−0.587	0.458
S10 Utilities	0.379^***^	−4.559^***^	0.183	0.381^***^	−1.381^**^	−4.265^***^	0.180

*Model 1: $r_{jt}=\alpha_{j}+\beta_{j}\cdot r_{mt}+\gamma_{j}\cdot \Delta i_{t}^{u}+\varepsilon_{jt}$, Model 2: $r_{jt}=\alpha_{j}+\beta_{jm}\cdot r_{mt}+\beta_{jr}\cdot \Delta r_{t}+\beta_{j\pi }\cdot \Delta E_{t}^{ORT}\left(\pi_{t,t+\text{1}}\right)+\varepsilon_{jt}$*.

*r_jt_ represents stock returns at time t for each sector j, r_mt_ is the return on the market portfolio, ${\Delta i}_{t}^{u}$ represents changes in nominal interest rates, Δr_t_ represents changes in real interest rates, $\Delta E_{t}^{ORT}\left(\pi_{t,t+\text{1}}\right)$ shows movements in expected inflation rates (orthogonalized), and finally, ε_t_ is the error term. The sample extends from Nov. 1989 to Feb. 2014 and the following regression has been estimated using SUR methodology*.

t-statistics in parentheses

* *p < 0.10*,

** *p < 0.05*,

*** *p < 0.01*.

Looking at model 1 for the overall sample period, the results confirms a noteworthy relationship between sector stock returns and unexpected changes in nominal interest rates as 6 of the 10 sectors have a statistically significant coefficient. Interestingly, the sign of this relationship is not always negative. Consumer Staples, Health Care, and Utilities are conventionally negative but Energy and Materials are marginally positive and Information Technology is significantly positive. Clearly, the positive coefficient for Information Technology is not due to mere chance. Moreover, the relation between stock returns and unexpected changes in nominal rates for the Information Technology sector remains significantly positive for the contraction and expansion sub-periods. This suggests that investors who seek protection from unanticipated interest rate changes can view an investment in a portfolio of Information Technology stocks as a natural hedge against interest rate risk.

Meanwhile, the conventionally negative relation between stock returns and unexpected changes in nominal interest rates for Consumer Staples, Health Care, and Utilities remain negative for the recession and expansion sub-periods but only the Consumer Staples coefficient remains highly significant in both sub-periods. Clearly, an investment in the Consumer Staples sector is subject to a significant amount of interest rate risk. Finally, there are two sectors without any significant relation between stock returns and unexpected changes in nominal interest rates for the entire sample but show significant coefficients, with opposite signs, for the contraction and expansion sub-periods. Specifically, Consumer Discretionary, and Industrials have the conventional inverse relation during contraction which turns positive during expansion suggesting that firms in these industries can pass on additional financing costs when economic conditions are robust.

When decomposing unexpected changes in the nominal rate of interest into unexpected changes in the real rate of interest and unexpected changes in the inflation rate (model 2), we discover comparable results for unanticipated changes in the real rate of interest but in this case, there are just four rather than six sectors that are statistically significant. Consumer Staples and Utilities have a significant inverse relation between stock returns and unexpected changes in the real rate of interest whereas Energy and Materials have a significant positive relation. However, none of these relations remains consistently significant and of the same sign for the contraction and expansion sub-periods with the exception of Energy. Even then the positive coefficient in the expansion sub-period is only marginally significant.

Similarly, the signs of the relation between stock returns and unanticipated inflation are not always negative. Specifically, we find significant negative coefficients for Consumer Staples, Health Care, and Utilities and one positive relation for Information Technology. However, only Consumer Staples has a consistent inverse relation for both economic sub-periods suggesting that unexpected changes in inflation are an important source of risk for investments in the Consumer Staples sector. Interestingly, stock returns in the Industrials sector are directly related to unanticipated inflation in expansion sub-period but are inversely related to unanticipated inflation in contraction sub-period suggesting that firms in this sector can pass on unexpected inflationary costs during robust economic conditions but are less able to do so during harder economic times.

In summary, we find that when there are significant relations between stock returns and unanticipated changes in nominal interest rates and their components, unanticipated changes in the real rate of interest and inflation, these relations are most commonly negative. The Consumer Staples industry sector shows this tendency most strongly as the relation between stock returns and unanticipated changes in the nominal interest rate as well as unanticipated changes in the inflation rate are significantly negative overall and in the contraction and expansion sub-periods. Even the relation between stock returns and unexpected changes in the real rate is negative but significantly so only for the contraction sub-period. Meanwhile we observe the contrary positive relation more rarely. The clearest example is the Information Technology sector. Specifically, while all the significant relations between stock returns in the Information Technology sector and unanticipated changes in nominal interest rate, real rate and inflation rate are always positive, they are consistently and significantly positive overall and in the in the contraction and expansion sub-periods only for unexpected changes in the nominal rate of interest. The next step is to see if we can discover more instances of these significant relationships as we further refine our analysis by examining more refined sub sector portfolios.

### Results at the sub-sector level

In the second step of our analysis, we estimate model 1 and 2 at the sub-sector level as defined in Supplementary Material Table A. Table 5 shows the number and percentage of sub-sectors that have a significant response of stock returns to unanticipated changes in each factor (nominal interest, real interest and inflation rate) and the average significant coefficient and the average positive and negative coefficients for each factor. Table 5A shows this information for the entire sample period while Tables 5B,C report this information for the contraction and expansion sub-periods, respectively.

**Table 5:** **Coefficients of sub-sector stock returns to variations in nominal interest rates (model 1) and real interest and expected inflation rates (model 2): % of significant exposure**.

**(A) TOTAL SAMPLE (FROM NOV. 1989 TO FEB. 2014)**		
**MODEL 1**		
***r**_*mt*_*	**Sub-sectors with signific. 10%**	**Average Coeff**.
Significant Coeff.	33 (100%)	1.011
Positive Coeff.	33 (100%)	1.011
Negative Coeff.	0	na
*${\Delta i}_{t}^{u}$*	**Sub-sectors with signific. 10%**	**Average Coeff**.
Significant Coeff.	14 (42.42%)	−0.545
Positive Coeff.	6 (18.18%)	2.499
Negative Coeff.	8 (24.24%)	−2.828
Average Ad. R^2^ = 45.21%		
**MODEL 2**		
***r**_*mt*_*	**Sub-sectors with signific. 10%**	**Average Coeff**.
Significant Coeff.	33 (100%)	1.011
Positive Coeff.	33 (100%)	1.011
Negative Coeff.	0	na
*Δ**r**_*t*_*	**Sub-sectors with signific. 10%**	**Average Coeff**.
Significant Coeff.	7 (21.21%)	−0.125
Positive Coeff.	3 (9.09%)	1.415
Negative Coeff.	4 (12.12%)	−1.280
$\Delta E_{t}^{ORT}\left(\pi_{t,t+1}\right)$	**Sub-sectors with signific. 10%**	**Average Coeff**.
Significant Coeff.	14 (42.42%)	−0.388
Positive Coeff.	6 (18.18%)	2.710
Negative Coeff.	8 (24.24%)	−2.712
Average Ad. *R*^2^ = 45.18%		
Total number of sub-sectors = 33		
**(B) CONTRACTION SUB-PERIOD**		
**MODEL 1**		
***r**_*mt*_*	**Sub-sectors with signific. 10%**	**Average Coeff**.
Significant Coeff.	33 (100%)	1.105
Positive Coeff.	33 (100%)	1.105
Negative Coeff.	0	na
*${\Delta i}_{t}^{u}$*	**Sub-sectors with signific. 10%**	**Average Coeff**.
Significant Coeff.	12 (36.36%)	−1.336
Positive Coeff.	3 (9.09%)	6.954
Negative Coeff.	9 (27.27%)	−4.099
Average Ad. *R*^2^ = 58.56%		
**MODEL 2**		
***r**_*mt*_*	**Sub-sectors with signific. 10%**	**Average Coeff**.
Significant Coeff.	33 (100%)	1.105
Positive Coeff.	33 (100%)	1.105
Negative Coeff.	0	na
*Δ**r**_*t*_*	**Sub-sectors with signific. 10%**	**Average Coeff**.
Significant Coeff.	6 (18.18%)	−0.526
Positive Coeff.	2 (6.06%)	2.708
Negative Coeff.	4 (12.12%)	−2.143
*$\Delta E_{t}^{ORT}\left(\pi_{t,t+1}\right)$*	**Sub-sectors with signific. 10%**	**Average Coeff**.
Significant Coeff.	11 (33.33%)	−0.422
Positive Coeff.	4 (12.12%)	6.945
Negative Coeff.	7 (21.21%)	−4.631
Average Ad. *R*^2^ = 58.32%		
Total number of sub-sectors = 33		
**(C) EXPANSION SUB-PERIOD**		
**MODEL 1**		
***r**_*mt*_*	**Sub-sectors with signific. 10%**	**Average Coeff**.
Significant Coeff.	32 (96.97%)	0.965
Positive Coeff.	32 (96.97%)	0.965
Negative Coeff.	0	na
*${\Delta i}_{t}^{u}$*	**Sub-sectors with signific. 10%**	**Average Coeff**.
Significant Coeff.	10 (30.30%)	−0.933
Positive Coeff.	4 (12.12%)	2.751
Negative Coeff.	6 (18.18%)	−3.389
Average Ad. *R*^2^ = 39.08%		
**MODEL 2**		
***r**_*mt*_*	**Sub-sectors with signific. 10%**	**Average Coeff**.
Significant Coeff.	32 (96.97%)	0.964
Positive Coeff.	32 (96.97%)	0.964
Negative Coeff.	0	na
*Δ**r**_*t*_*	**Sub-sectors with signific. 10%**	**Average Coeff**.
Significant Coeff.	7 (21.21%)	0.483
Positive Coeff.	5 (15.15%)	1.269
Negative Coeff.	2 (6.06%)	−1.482
$\Delta E_{t}^{ORT}\left(\pi_{t,t+1}\right)$	**Sub-sectors with signific. 10%**	**Average Coeff**.
Significant Coeff.	9 (27.27%)	−0.924
Positive Coeff.	3 (9.09%)	3.907
Negative Coeff.	6 (18.18%)	−3.339
Average Ad. *R*^2^ = 38.95%		
Total number of sub-sectors = 33		

*Model 1: $r_{jt}=\alpha_{j}+\beta_{j}\cdot r_{mt}+\gamma_{j}\cdot \Delta i_{t}^{u}+\varepsilon_{jt}$; Model 2: $r_{jt}=\alpha_{j}+\beta_{jm}\cdot r_{mt}+\beta_{jr}\cdot \Delta r_{t}+\beta_{j\pi }\cdot \Delta E_{t}^{ORT}\left(\pi_{t,t+\text{1}}\right)+\varepsilon_{jt}$*.

r_jt_ represents stock returns at time t for each industry j, r_mt_ is the return on the market portfolio, ${\Delta i}_{t}^{u}$ represents changes in nominal interest rates, Δr_t_ represents changes in real interest rates, $\Delta E_{t}^{ORT}\left(\pi_{t,t+\text{1}}\right)$ shows movements in expected inflation rates (orthogonalized) and, finally, ε_t_ is the error term. The sample extends from Nov. 1989 to Feb. 2014 and the following regression has been estimated using SUR methodology. t-statistics in parentheses ^*^ p < 0.10, ^**^ p < 0.05, ^***^ p < 0.01.

For both models, we find a positive and significant market beta for the total sample and for the expansion and contraction sub-periods for all sub-sectors with just one exception. There are <100% sub-sectors with a statistically significant positive market beta during the expansion sub-period because the beta for Construction Materials, while positive, is statistically insignificant. The average beta is close to the theoretical beta of 1, being a little higher in the contraction sub-period and a little lower in the expansion sub-period. For the overall period, betas range between about 0.4 for Electric Utilities and 1.5 for Semiconductors and Semiconductor Equipment. For the sake of brevity, we do not report the coefficients for each of the 33 sub-sectors. They are available from the corresponding author upon request.

The average significant sub-sector coefficients, along with the average of the significant positive and negative coefficients are shown in column 3 of Table 5. The average relation between stock returns and unexpected changes in the nominal interest rate (model 1) and unexpected changes in the real interest and inflation rates (model 2) are negative for the overall period and for the contraction and expansion sub-periods with just one exception. Specifically, in Table 5C the average coefficient for unexpected changes in the real rate of interest is a positive 0.483 for the expansion sub-period. Moreover, when a coefficient is significant, it is most often negative, again except for the expansion sub-period for unexpected changes in the real rate of interest. Specifically, Table 5C, column 2 shows that five of the seven sub-sectors have a significant positive relation between stock returns and unexpected changes in the real rate of interest.

Clearly, the overall results are consistent with most of the prior literature as the relations between stock returns and unexpected changes in the rate of inflation are most often negative. Specifically, column 2 shows that around 42, 33, and 27% of the sub-sectors for the total sample, contraction, and expansion sub-periods, respectively, have stock returns that are significantly and negatively related to unexpected changes in the inflation rate.

Nevertheless, there are some exceptions to the conventionally inverse relations. For instance, Table 5A, column 2 reports that there are six sub-sectors that have a significant positive relation between stock returns and unexpected changes in the nominal rate of interest in the overall period. In addition, we find three contrary positive relations for unexpected changes in the real rate of interest and six contrary positive relations for unexpected changes in inflation rate for the overall sample period. Breaking down the results by sub-period, we observe that with a smaller sample size, there are fewer statistically significant coefficients. During the contraction sub-period, there are more instances of inverse relations and during the expansion sub-period, there are proportionally more instances of positive relations suggesting that on average companies find it easier to pass on unexpected costs during expansions.

In summary, we find that on average, the relation between stock returns and unanticipated changes in the nominal rates of interest (model 1) and unanticipated changes in the real rate of interest and the inflation rate (model 2) are negative. This result is consistent with the literature. However, as we saw at the more aggregate sector level, we continue to find contrary positive relations at the sub-sector level. This motivates us to examine individual industries to see if we can find exceptional industries where investments in these industries can form a natural hedge against sources interest rate and inflation risk.

### Results at an industry level

As a last step, we regress models 1 and 2 at the industry level. We again examine the relations for the total sample, contraction, and expansion sub-periods and obtain some remarkable results. Tables 6A1,A2 shows the results for model 1 and 2, respectively, for the overall period and Tables 6B1,B2,C1,C2 show the results for model 1 and 2 for the contraction and expansion sub-periods, respectively. All panels present the information in the same way. For instance, Table 6A1, columns 2–5 show by sector the number of industries, the proportion that have a significant response to each factor and the number industries that have a positive and a negative response to each factor, respectively. Column 6 reports the average significant coefficient for the sector and the range of coefficient values by sector while columns 7 and 8 reports the size of the average positive and negative coefficients.

**Table 6:** **Coefficients of industry stock returns to variations in nominal interest rates (model 1) and real interest and expected inflation rates (model 2): Significant industry sensitivity**.

**(A1) MODEL 1 TOTAL SAMPLE (FROM NOV. 1989 TO FEB. 2014)**							
**Model 1**		**Industries with signific. 10%**			**Average Coeff**.		
***r_*mt*_***	**Nr. Ind**	**Signif. Coeff**.	**Posit. Coeff**.	**Negat. Coeff**.	**Signif. Coeff. (range)**	**Posit. Coeff**.	**Negat. Coeff**.
Industries of S1 Consum. Discretionary	16	16/16	16	0	1.090 (0.691, 1.446)	1.090	na
Industries of S2 Consumer Staples	9	9/9	9	0	0.649 (0.422, 1.054)	0.649	na
Industries of S3 Energy	7	7/7	7	0	1.011 (0.656, 1.328)	1.011	na
Industries of S4 Financials	11	11/11	11	0	1.351 (0.834, 2.103)	1.351	na
Industries of S5 Health Care	5	5/5	5	0	0.731 (0.649, 0.795)	0.731	na
Industries of S6 Industrials	12	12/12	12	0	1.062 (0.752, 1.498)	1.062	na
Industries of S7 Inform. Technology	9	9/9	9	0	1.480 (0.906, 1.833)	1.480	na
Industries of S8 Materials	12	12/12	12	0	1.097 (0.354, 1.638)	1.097	na
Industries of S9 Telecommunications	1	1/1	1	0	0.791 (0.791, 0.791)	0.791	na
Industries of S10 Utilities	0	na	na	na	na	na	na
Total number of industries	82	82	82	0			
		**Industries with signific. 10%**			**Average Coeff**.		
*${\Delta i}_{t}^{u}$*	**Nr. Ind**	**Signif. Coeff**.	**Posit. Coeff**.	**Negat. Coeff**.	**Signif. Coeff. (range)**	**Posit. Coeff**.	**Negat. Coeff**.
Industries of S1 Consum. Discretionary	16	4/16	3	1	1.496 (−2.662, 3.528)	2.881	−2.662
Industries of S2 Consumer Staples	9	6/9	0	6	−2.797 (−5.293, −1.664)	na	−2.797
Industries of S3 Energy	7	2/7	2	0	7.256 (5.771, 8.741)	7.256	na
Industries of S4 Financials	11	4/11	2	2	0.578 (−5.067, 6.754)	5.007	−3.853
Industries of S5 Health Care	5	2/5	1	1	1.740 (−2.327, 5.808)	5.808	−2.327
Industries of S6 Industrials	12	1/12	0	1	−4.154 (−4.154, −4.154)	na	−4.154
Industries of S7 Inform. Technology	9	5/9	5	0	3.714 (2.596, 6.491)	3.714	na
Industries of S8 Materials	12	3/12	2	1	0.399 (−6.365, 5.235)	3.780	−6.365
Industries of S9 Telecommunications	1	1/1	0	1	−1.908 (−1.908, −1.908)	na	−1.908
Industries of S10 Utilities	0	na	na	na	na	na	na
Total number of industries	82	28	15	13			
		Average Ad. *R*^2^ = 40.81%					
**(A2) MODEL 2 TOTAL SAMPLE (FROM NOV. 1989 TO FEB. 2014)**							
**Model 2**		**Industries with signific. 10%**			**Average Coeff**.		
***r_*mt*_***	**Nr. Ind**	**Signif. Coeff**.	**Posit. Coeff**.	**Negat. Coeff**.	**Signif. Coeff. (range)**	**Posit. Coeff**.	**Negat. Coeff**.
Industries of S1 consum. discretionary	16	16/16	16	0	1.089 (0.705, 1.452)	1.089	na
Industries of S2 consumer staples	9	9/9	9	0	0.649 (0.426, 1.056)	0.649	na
Industries of S3 energy	7	7/7	7	0	1.008 (0.650, 1.326)	1.008	na
Industries of S4 financials	11	11/11	11	0	1.351 (0.834, 2.102)	1.351	na
Industries of S5 health care	5	5/5	5	0	0.731 (0.647, 0.797)	0.731	na
Industries of S6 industrials	12	12/12	12	0	1.062 (0.754, 1.502)	1.062	na
Industries of S7 inform. technology	9	9/9	9	0	1.475 (0.905, 1.826)	1.475	na
Industries of S8 materials	12	12/12	12	0	1.096 (0.342, 1.637)	1.096	na
Industries of S9 telecommunications	1	1/1	1	0	0.792 (0.792, 0.792)	0.792	na
Industries of S10 Utilities	0	na	na	na	na	na	na
Total number of industries	82	82	82	0			
		**Industries with signific. 10%**			**Average Coeff**.		
*Δ**r**_*t*_*	**Nr. Ind**	**Signif. Coeff**.	**Posit. Coeff**.	**Negat. Coeff**.	**Signif. Coeff. (range)**	**Posit. Coeff**.	**Negat. Coeff**.
Industries of S1 Consum. Discretionary	16	1/16	1	0	1.356 (1.356, 1.356)	1.356	na
Industries of S2 Consumer Staples	9	3/9	0	3	−1.295 (−1.430, −1.119)	na	−1.295
Industries of S3 Energy	7	2/7	2	0	1.647 (1.227, 2.066)	1.647	na
Industries of S4 Financials	11	2/11	1	1	−0.138 (−1.415, 1.141)	1.141	−1.415
Industries of S5 Health Care	5	0/5	0	0	na	na	na
Industries of S6 Industrials	12	4/12	1	3	−0.975 (−2.523, 1.116)	1.116	−1.672
Industries of S7 Inform. Technology	9	2/9	2	0	2.843 (2.537, 3.150)	2.843	na
Industries of S8 Materials	12	2/12	2	0	2.972 (2.252, 3.691)	2.972	na
Industries of S9 Telecommunications	1	0/1	0	0	na	na	na
Industries of S10 Utilities	0	na	na	na	na	na	na
Total number of industries	82	16	9	7			
		**Industries with signific. 10%**			**Average Coeff**.		
$\Delta E_{t}^{ORT}\left(\pi_{t,t+1}\right)$	**Nr. Ind**	**Signif. Coeff**.	**Posit. Coeff**.	**Negat. Coeff**.	**Signif. Coeff. (range)**	**Posit. Coeff**.	**Negat. Coeff**.
Industries of S1 Consum. Discretionary	16	5/16	2	3	−0.297 (−3.590, 4.549)	3.486	−2.819
Industries of S2 Consumer Staples	9	4/9	0	4	−3.009 (−5.181, −1.888)	na	−3.009
Industries of S3 Energy	7	2/7	2	0	7.193 (6.007, 8.379)	7.193	na
Industries of S4 Financials	11	3/11	1	2	−1.512 (−4.984, 3.046)	3.046	−3.791
Industries of S5 Health Care	5	2/5	1	1	1.856 (−2.293, 6.004)	6.004	−2.293
Industries of S6 Industrials	12	2/12	1	1	−0.359 (−3.619, 2.902)	2.902	−3.619
Industries of S7 Inform. Technology	9	2/9	2	0	4.272 (2.804, 5.741)	4.272	na
Industries of S8 Materials	12	3/12	2	1	−0.897 (−8.356, 2.867)	2.833	−8.356
Industries of S9 Telecommunications	1	0/1	0	0	na	na	na
Industries of S10 Utilities	0	na	na	na	na	na	na
Total number of industries	82	23	11	12			
		Average Ad. *R*^2^ = 40.82%					
**(B1) MODEL 1 CONTRACTION SUB-PERIOD**							
**Model 1**		**Industries with signific. 10%**			**Average Coeff**.		
***r_*mt*_***	**Nr. Ind**	**Signif. Coeff**.	**Posit. Coeff**.	**Negat. Coeff**.	**Signif. Coeff. (range)**	**Posit. Coeff**.	**Negat. Coeff**.
Industries of S1 Consum. Discretionary	16	16/16	16	0	1.310 (0.448, 2.116)	1.310	na
Industries of S2 Consumer Staples	9	9/9	9	0	0.684 (0.451, 1.386)	0.684	na
Industries of S3 Energy	7	7/7	7	0	0.969 (0.605, 1.290)	0.969	na
Industries of S4 Financials	11	11/11	11	0	1.601 (0.638, 2.553)	1.601	na
Industries of S5 Health Care	5	5/5	5	0	0.664 (0.410, 0.856)	0.664	na
Industries of S6 Industrials	12	12/12	12	0	1.304 (0.904, 2.126)	1.304	na
Industries of S7 Inform. Technology	9	9/9	9	0	1.419 (0.962, 1.819)	1.419	na
Industries of S8 Materials	12	11/12	11	0	1.403 (0.719, 2.130)	1.403	na
Industries of S9 Telecommunications	1	1/1	1	0	0.561 (0.561, 0.561)	0.561	na
Industries of S10 Utilities	0	na	na	na	na	na	na
Total number of industries	82	81	81	0			
		**Industries with signific. 10%**			**Average Coeff**.		
*${\Delta i}_{t}^{u}$*	**Nr. Ind**	**Signif. Coeff**.	**Posit. Coeff**.	**Negat. Coeff**.	**Signif. Coeff. (range)**	**Posit. Coeff**.	**Negat. Coeff**.
Industries of S1 Consum. Discretionary	16	6/16	0	6	−5.790 (−8.784, −3.734)	na	−5.790
Industries of S2 Consumer Staples	9	2/9	0	2	−3.731 (−4.059, −3.403)	na	−3.731
Industries of S3 Energy	7	5/7	5	0	12.775 (9.737, 17.048)	12.775	na
Industries of S4 Financials	11	4/11	0	4	−7.095 (−9.574, −4.173)	na	−7.095
Industries of S5 Health Care	5	2/5	1	1	2.451 (−2.838, 7.741)	7.741	−2.838
Industries of S6 Industrials	12	4/12	0	4	−6.360 (−11.676, −3.017)	na	−6.360
Industries of S7 Inform. Technology	9	3/9	3	0	6.252 (4.949, 8.512)	6.252	na
Industries of S8 Materials	12	4/12	2	2	0.606 (−8.414, 8.019)	7.689	−6.477
Industries of S9 Telecommunications	1	1/1	0	1	−3.963 (−3.963, −3.963)	na	−3.963
Industries of S10 Utilities	0	na	na	na	na	na	na
Total number of industries	82	31	11	20			
		Average Ad. R^2^ = 54.95%					
**(B2) MODEL 2 CONTRACTION SUB-PERIOD**							
**Model 2**		**Industries with signific. 10%**			**Average Coeff**.		
***r_*mt*_***	**Nr. Ind**	**SignifCoeff**.	**Posit. Coeff**.	**Negat. Coeff**.	**Signif. Coeff. (range)**	**Posit. Coeff**.	**Negat. Coeff**.
Industries of S1 consum. discretionary	16	16/16	16	0	1.310 (0.437, 2.124)	1.310	na
Industries of S2 consumer staples	9	9/9	9	0	0.684 (0.447, 1.389)	0.684	na
Industries of S3 energy	7	7/7	7	0	0.965 (0.600, 1.287)	0.965	na
Industries of S4 financials	11	11/11	11	0	1.602 (0.644, 2.536)	1.602	na
Industries of S5 health care	5	5/5	5	0	0.664 (0.410, 0.855)	0.664	na
Industries of S6 industrials	12	12/12	12	0	1.306 (0.906, 2.134)	1.306	na
Industries of S7 inform. technology	9	9/9	9	0	1.414 (0.962, 1.816)	1.414	na
Industries of S8 materials	12	11/12	11	0	1.402 (0.719, 2.136)	1.402	na
Industries of S9 telecommunications	1	1/1	1	0	0.562 (0.562, 0.562)	0.562	na
Industries of S10 utilities	0	na	na	na	na	na	na
Total number of industries	82	81	81	0			
		**Industries with signific. 10%**			**Average Coeff**.		
***Δr_*t*_***	**Nr. Ind**	**Signif. Coeff**.	**Posit. Coeff**.	**Negat. Coeff**.	**Signif. Coeff. (range)**	**Posit. Coeff**.	**Negat. Coeff**.
Industries of S1 consum. discretionary	16	2/16	0	2	−2.613 (−3.003, −2.224)	na	−2.613
Industries of S2 consumer staples	9	2/9	0	2	−2.127 (−2.267, −1.986)	na	−2.127
Industries of S3 energy	7	6/7	6	0	3.693 (1.582, 5.743)	3.693	na
Industries of S4 financials	11	2/11	0	2	−2.241 (−2.407, −2.075)	na	−2.241
Industries of S5 health care	5	0/5	0	0	na	na	na
Industries of S6 Industrials	12	5/12	1	4	−2.490 (−4.979, 1.066)	1.066	−3.379
Industries of S7 inform. technology	9	3/9	3	0	3.171 (1.961, 5.159)	3.171	na
Industries of S8 materials	12	1/12	1	0	5.139 (5.139, 5.139)	5.139	na
Industries of S9 telecommunications	1	0/1	0	0	na	na	na
Industries of S10 utilities	0	na	na	na	na	na	na
Total number of industries	82	21	11	10			
		**Industries with signific. 10%**			**Average Coeff**.		
$\Delta E_{t}^{ORT}\left(\pi_{t,t+1}\right)$	**Nr. Ind**	**Signif. Coeff**.	**Posit. Coeff**.	**Negat. Coeff**.	**Signif. Coeff. (range)**	**Posit. Coeff**.	**Negat. Coeff**.
Industries of S1 consum. discretionary	16	6/16	0	6	−5.795 (−8.900, −4.215)	na	−5.795
Industries of S2 consumer staples	9	2/9	0	2	−3.510 (−3.781, −3.239)	na	−3.510
Industries of S3 energy	7	5/7	5	0	12.575 (9.598, 16.967)	12.575	na
Industries of S4 financials	11	3/11	0	3	−8.063 (−9.734, −6.010)	na	−8.063
Industries of S5 health care	5	2/5	1	1	2.595 (−2.723, 7.913)	7.913	−2.723
Industries of S6 industrials	12	5/12	0	5	−5.489 (−10.823, −2.129)	na	−5.489
Industries of S7 inform. technology	9	3/9	3	0	5.623 (4.589, 7.297)	5.623	na
Industries of S8 materials	12	3/12	1	2	−2.456 (−9.546, 7.368)	7.368	−7.368
Industries of S9 telecommunications	1	1/1	0	1	−3.824 (−3.824, −3.824)	na	−3.824
Industries of S10 utilities	0	na	na	na	na	na	na
Total number of industries	82	30	10	20			
		Average Ad. *R*^2^ = 54.88%					
**(C1) MODEL 1 EXPANSION SUB-PERIOD**							
**Model 1**		**Industries with signific. 10%**			**Average Coeff**.		
***r_*mt*_***	**Nr. Ind**	**Signif. Coeff**.	**Posit. Coeff**.	**Negat. Coeff**.	**Signif. Coeff. (range)**	**Posit. Coeff**.	**Negat. Coeff**.
Industries of S1 consum. discretionary	16	16/16	16	0	1.011 (0.629, 1.425)	1.011	na
Industries of S2 consumer staples	9	9/9	9	0	0.620 (0.343, 0.871)	0.620	na
Industries of S3 energy	7	7/7	7	0	1.057 (0.679, 1.859)	1.057	na
Industries of S4 financials	11	11/11	11	0	1.150 (0.788, 1.554)	1.150	na
Industries of S5 health care	5	5/5	5	0	0.821 (0.680, 1.125)	0.821	na
Industries of S6 industrials	12	12/12	12	0	0.931 (0.683, 1.159)	0.931	na
Industries of S7 inform. technology	9	9/9	9	0	1.528 (0.810, 1.981)	1.528	na
Industries of S8 materials	12	12/12	12	0	0.998 (0.370, 1.358)	0.998	na
Industries of S9 telecommunications	1	1/1	1	0	0.931 (0.931, 0.931)	0.931	na
Industries of S10 utilities	0	na	na	na	na	na	na
Total number of industries	82	82	82	0			
		**Industries with signific. 10%**			**Average Coeff**.		
*${\Delta i}_{t}^{u}$*	**Nr. Ind**	**Signif. Coeff**.	**Posit. Coeff**.	**Negat. Coeff**.	**Signif. Coeff. (range)**	**Posit. Coeff**.	**Negat. Coeff**.
Industries of S1 consum. discretionary	16	3/16	3	0	3.086 (1.991, 4.295)	3.086	na
Industries of S2 consumer staples	9	4/9	0	4	−3.738 (−6.800, −2.520)	na	−3.738
Industries of S3 energy	7	0/7	0	0	na	na	na
Industries of S4 financials	11	4/11	3	1	4.974 (−4.594, 16.511)	8.163	−4.594
Industries of S5 health care	5	2/5	0	2	−1.949 (−2.089, −1.810)	na	−1.949
Industries of S6 industrials	12	3/12	3	0	2.823 (1.809, 4.143)	2.823	na
Industries of S7 inform. technology	9	3/9	3	0	4.437 (2.643, 7.290)	4.437	na
Industries of S8 materials	12	5/12	4	1	1.588 (−5.602, 3.940)	3.386	−5.602
Industries of S9 telecommunications	1	0/1	0	0	na	na	na
Industries of S10 utilities	0	na	na	na	na	na	na
Total number of industries	82	24	16	8			
		Average Ad. *R*^2^ = 34.43%					
**(C2) MODEL 2 EXPANSION SUB-PERIOD**							
**Model 2**		**Industries with signific. 10%**			**Average Coeff**.		
***r_*mt*_***	**Nr. Ind**	**SignifCoeff**.	**Posit. Coeff**.	**Negat. Coeff**.	**Signif. Coeff. (range)**	**Posit. Coeff**.	**Negat. Coeff**.
Industries of S1 consum. discretionary	16	16/16	16	0	1.014 (0.650, 1.398)	1.014	na
Industries of S2 consumer staples	9	9/9	9	0	0.620 (0.359, 0.872)	0.620	na
Industries of S3 energy	7	7/7	7	0	1.067 (0.670, 1.927)	1.067	na
Industries of S4 financials	11	11/11	11	0	1.140 (0.785, 1.568)	1.140	na
Industries of S5 health care	5	5/5	5	0	0.821 (0.687, 1.128)	0.821	na
Industries of S6 industrials	12	12/12	12	0	0.930 (0.682, 1.159)	0.930	na
Industries of S7 inform. technology	9	9/9	9	0	1.524 (0.806, 1.966)	1.524	na
Industries of S8 materials	12	12/12	12	0	0.997 (0.356, 1.346)	0.997	na
Industries of S9 telecommunications	1	1/1	1	0	0.934 (0.934, 0.934)	0.934	na
Industries of S10 utilities	0	na	na	na	na	na	na
Total number of industries	82	82	82	0			
		**Industries with signific. 10%**			**Average Coeff**.		
***Δr_*t*_***	**Nr. Ind**	**Signif. Coeff**.	**Posit. Coeff**.	**Negat. Coeff**.	**Signif. Coeff. (range)**	**Posit. Coeff**.	**Negat. Coeff**.
Industries of S1 consum. discretionary	16	4/16	3	1	0.374 (−3.487, 2.022)	1.661	−3.487
Industries of S2 consumer staples	9	0/9	0	0	na	na	na
Industries of S3 energy	7	1/7	1	0	1.199 (1.199, 1.199)	1.199	na
Industries of S4 financials	11	2/11	2	0	3.679 (2.148, 5.210)	3.679	na
Industries of S5 health care	5	0/5	0	0	na	na	na
Industries of S6 industrials	12	1/12	1	0	0.919 (0.919, 0.919)	0.919	na
Industries of S7 inform. technology	9	1/9	1	0	3.580 (3.580, 3.580)	3.580	na
Industries of S8 materials	12	2/12	2	0	2.666 (1.969, 3.362)	2.666	na
Industries of S9 telecommunications	1	0/1	0	0	na	na	na
Industries of S10 utilities	0	na	na	na	na	na	na
Total number of industries	82	11	10	1			
		**Industries with signific. 10%**			**Average Coeff**.		
$\Delta E_{t}^{ORT}\left(\pi_{t,t+1}\right)$	**Nr. Ind**	**Signif. Coeff**.	**Posit. Coeff**.	**Negat. Coeff**.	**Signif. Coeff. (range)**	**Posit. Coeff**.	**Negat. Coeff**.
Industries of S1 consum. discretionary	16	3/16	2	1	1.755 (−2.500, 5.048)	3.882	−2.500
Industries of S2 consumer staples	9	3/9	0	3	−4.448 (−7.320, −2.927)	na	−4.448
Industries of S3 energy	7	0/7	0	0	na	na	na
Industries of S4 financials	11	3/11	2	1	4.650 (−5.052, 15.356)	9.501	−5.052
Industries of S5 health care	5	2/5	0	2	−2.262 (−2.367, −2.157)	na	−2.262
Industries of S6 industrials	12	3/12	3	0	2.991 (1.899, 4.009)	2.991	na
Industries of S7 inform. technology	9	2/9	2	0	3.221 (2.564, 3.878)	3.221	na
Industries of S8 materials	12	5/12	3	2	0.371 (−7.525, 4.558)	3.771	−4.730
Industries of S9 telecommunications	1	0/1	0	0	na	na	na
Industries of S10 utilities	0	na	na	na	na	na	na
Total number of industries	82	21	12	9			
		Average Ad. *R*^2^ = 34.32%					

*Model 1: $r_{jt}=\alpha_{j}+\beta_{j}\cdot r_{mt}+\gamma_{j}\cdot \Delta i_{t}^{u}+\varepsilon_{jt}$; Model 2: $r_{jt}=\alpha_{j}+\beta_{jm}\cdot r_{mt}+\beta_{jr}\cdot \Delta r_{t}+\beta_{j\pi }\cdot \Delta E_{t}^{ORT}\left(\pi_{t,t+\text{1}}\right)+\varepsilon_{jt}$*.

r_jt_ represents stock returns at time t for each industry j, r_mt_ is the return on the market portfolio, ${\Delta i}_{t}^{u}$ represents changes in nominal interest rates, Δr_t_ represents changes in real interest rates, $\Delta E_{t}^{ORT}\left(\pi_{t,t+\text{1}}\right)$ shows movements in expected inflation rates (orthogonalized) and, finally, ε_t_ is the error term. The sample extends from Nov. 1989 to Feb. 2014 and the following regression has been estimated using SUR methodology. t-statistics in parentheses ^*^ p < 0.10, ^**^ p < 0.05, ^***^ p < 0.01.

For both models in all six panels from A1 to C2, all industries exhibit positive and significant market betas for the overall sample and for the expansion and contraction sub-periods with just one interesting exception. While all of the industry betas during the contraction sub-period in the Materials sub-sector are positive for both models, Tables 6B1,B2 show that only 11 of 12 industries have significant betas. The exceptional industry is Gold, long rumored to be an industry that can provide a safe haven during recessions.

Model 1 (in Table 6A1) reports that at the industry level, there are more instances of contrary positive relations between stock returns and unanticipated changes in nominal interest rates. In fact, the average industry weighted significant coefficient is positive for six of the nine sectors and only three have significant negative average coefficients. The Utilities industry sub-sector is not segmented into industries by Bloomberg so we can conduct our individual industry analysis only within the remaining nine industry sub-sectors. The sectors with the highest number of industries with significant coefficients are Consumer Staples with an average significant coefficient of −2.8 and Information Technology with an average significant coefficient of 3.7. Meanwhile in the Industrials sector, only 1 of 12 industries, namely Building Products, has a significant relation to unexpected changes in nominal interest rates with a coefficient of −4.15. Industries in the Energy sector exhibit the highest average significant response to unexpected changes in the nominal rate of interest (7.256) whereas industries within the Materials and Financials sectors are the least sensitive to unexpected changes in the nominal rate of interest at 0.399 and 0.578, respectively. The sectors with the most heterogeneous industries are Financials, Health Care and Materials as the range of significant coefficients is very large. In contrast, the most homogeneous industry is Consumer Staples where six of nine industries have a significant negative relation between stock returns and unanticipated changes in nominal interest rates.

In Table 6B1 we observe that the results of the contraction sub-period is mostly similar to the total sample but with a few peculiarities. First, the stock returns of more industries are inversely related to unanticipated changes in nominal interest rates to the point where Consumer Discretionary and Financials sectors now join the previous three industry sectors that have an average negative significant coefficient. Second, three more industries in the Energy sector and the Industrials sector now have a significant relation between stock returns and unanticipated changes in nominal interest rates. However, the three additional industries for the Industrials sector are all negatively and the three industries for the Energy sector are all positively related to unanticipated changes in the nominal rate of interest. Meanwhile, it is remarkable that two fewer industries in the Information Technology sector exhibit a significant relation between stock returns and nominal interest rate changes in the contraction sub-period and for the remaining significant Information Technology industries, the coefficients become more positive. Third, in general, we observe that stock returns are more responsive to unexpected changes in nominal interest rates, irrespective of the sign, during the contraction sub-period. Moreover, it is noteworthy that Health Care and Materials are again the sectors with the most heterogeneous response to unexpected changes in the nominal rate of interest and the range of significant values are even larger during the contraction sub-period.

In the expansion sub-period reported in Table 6C1, we find that compared to the overall sample, there are four fewer industries that have a significant relation between stock returns and unexpected changes in nominal interest rate. In fact, the Energy and Telecommunication sectors do not have even one industry that has a significant relation between stock returns and unexpected change in nominal interest rates. The range of significant coefficients is typically smaller as well. In terms of absolute values of the coefficients, stock returns of industries in the Financial sectors have the largest average response (nearly 5) to unexpected changes in the nominal rate of interest whereas industries in the Materials sector have the lowest average response (nearly 1.6) to unexpected changes in the nominal rate of interest.

There is an interest phenomenon contained within these results. Stock returns for the Diversified Metals and Mining industry (within the Materials sector) have a positive and significant relation between stock returns and unexpected changes in nominal interest rates for the overall, contraction and expansion sub-periods. This suggests that an investment in these industries can form a natural safe haven against unexpected changes in the nominal interest rate.

Meanwhile, Model 2 Tables 6A2,B2,C2 provides the following interesting observations. First, the stock returns of most industries have no significant relation with unexpected changes in the real rate of interest. For instance, in the overall period, only 16 of 82 industries have a significant coefficient and independent of the sample period, the stock returns of all industries in the Health Care sector does not have a significant relation to unexpected changes in the real rate of interest. There are a few more industries with a significant relation between stock returns and unexpected changes in the real rate of interest in the contraction sub-period and a few less in the expansion sub-period, 21 and 11, respectively. Clearly, the stock returns of most industries do not respond to unexpected changes in the real rate of interest.

However, within these general results we find three industries, one each in the Energy, Industrials and Materials sectors, have a consistently significant, and positive, relation between stock returns and unexpected changes in the real rate of interest. Specifically, we find that stock returns in the Integrated Oil and Gas, Commercial Services, and Supplies and Diversified Metals and Mining industries have a consistently positive relation with unexpected changes in the real rate of interest for the overall, contraction, and expansion sub-periods. This suggests that investors can find that an investment in these industries can provide some insulation from unexpected changes in the real rate of interest.

We find that the stock returns of many industries respond to unexpected changes in the inflation rate. Overall, 23 of 82 industries respond significantly to unexpected changes in inflation, while during the contraction sub-period the number of significant relations rises to 30 and during expansion the number of significant relations falls only slightly to 21. The stock returns for industries in the Energy sector exhibit the highest average response to unexpected changes in the inflation rate for the total sample (7.19) and contraction sub-period (12.58) whereas firms in the Financial sectors have the highest average response in the expansion sub-period (4.65). In contrast, industries in the Consumer Discretionary sector have the lowest average response to unexpected changes in the inflation rate for the total sample (−0.29) while industries in the Materials sector have the lowest response in the contraction and the expansion sub-periods, −2.46 and 0.37, respectively.

On average, the majority of sectors, most notably Consumer Discretionary, Consumer Staples, Financials, Industrials, and Materials, have an industry weighted negative significant relation between stock returns and unexpected inflation. While overall, and in some of the sub-periods, we can find industries with a significant positive relation between stock returns and unexpected inflation, we are unable to find an industry that has a consistently positive relation with unexpected inflation. However, we do find that stock returns in the Household Durables, Pharmaceuticals, and Gold industries have a negative relation to unanticipated inflation in the overall sample and in the contraction and expansion sub-periods suggesting that stocks in these industries are exposed to significant inflation risk.

### Overall results

As mentioned previously, according to most of literature, the response of stock returns to changes in nominal and real interest rates is usually negative. Our results generally agree with these previous findings. Also, like Booth and Officer (1985), Bae (1990), Jareño (2008), Ferrando et al. (2015), and Jareño et al. (2016), we find that some financial (as well as non-financial) sectors have insignificant relations. However, we also find some contrary results when examining the relations by sector, sub-sector, and industry. We find that three industries, specifically Integrated Oil and Gas, Commercial Services and Supplies, and Diversified Metals and Mining have a consistent significant positive relation between stock returns and unexpected changes in real interest rates while one industry, Diversified Metals and Mining, has a significant consistently positive relation between stock returns and unexpected changes in nominal interest rates. These positive relations suggest that long investments in portfolios of stocks in these particular industries can form a safe haven from unanticipated changes in nominal and real interest rates. Moreover, we find that Gold has an insignificant beta during recessionary conditions hinting that investments in the Gold industry can indeed be a safe haven during recessions. Interestingly, we find that three industries, specifically Household Durables, Pharmaceuticals, and Gold have a negative relation to unanticipated inflation in the overall sample and in the contraction and expansion sub-periods suggesting that these three industries are particularly exposed to inflation risk. It is remarkable that stock returns are inversely related to unexpected inflation for the Gold industry, thereby damaging the image of Gold as a hedge against inflation. Therefore, investor behavior seems to be quite different over time (according to the business cycle) and by sector.

## Discussion

Many studies have analyzed the sensitivity of stock returns to changes in nominal interest rates (Sweeney and Warga, 1986; Hevert et al., 1998a,b; O'Neal, 1998; Oertmann et al., 2000; Fraser et al., 2002; Tessaromatis, 2003; Jareño, 2006, 2008; Ferrer et al., 2010), finding a negative and significant relationship between stock returns and unexpected changes in nominal interest rates. We too examine this relationship but at the sector, sub-sector, and industry levels for both contraction and expansion sub-periods as well as for the overall sample period. In general, we find significant and negative relationship between stock returns and unexpected changes in nominal interest rates. Nevertheless, we observe important exceptions where some of these relations are insignificant and other relations that are consistently positive, even at the level of an individual industry in the case of the Diversified Metals and Mining industry.

At the sector level, we find that the most vulnerable sector to fluctuations in 10-year government bond yields are Utilities, so regulated and seriously indebted sectors seem to be the most interest rate sensitive, particularly in the expansion sub-period. Also, we note that Consumer Discretionary and Industrials have the conventional inverse relation between stock returns and unanticipated changes in the nominal rate of interest during the contraction sub-period that turns positive during the expansion sub-period so that for the overall period, there is no significant relation. This suggests that firms in these industries can pass on additional financing costs when economic conditions are robust.

In order to deepen in our analysis, we decompose unexpected changes in the nominal interest rate into unexpected changes in the real interest and inflation rates. In general, the stock returns by sector, sub-sector and industry are inversely related to unexpected changes in the real interest rate movements, and unexpected changes in the inflation rate overall and more so in the contraction than in expansion sub-period. However, it is unusual to find industries with a consistent negative relation between stock returns and unanticipated changes in the real interest rate and the inflation rate. There are three exceptions however. Evidently, inflation is an important source of risk for investments in Household Durables, Pharmaceuticals and Gold industries as they have a negative relation to unanticipated inflation in the overall sample and in the contraction and expansion sub-periods.

It is remarkable that stock returns are inversely related to unexpected inflation for the Gold industry, thereby damaging the image of Gold as a hedge against inflation. Another interesting result is that the stock returns in the Gold industry are not significantly related to the market return during contraction economic sub-periods thereby bolstering Gold's reputation as a safe haven during recessionary conditions.

Interestingly, we find that investments in three industries, specifically Integrated Oil and Gas, Commercial Services and Supplies, and Diversified Metals and Mining can provide a safe haven against unexpected changes in the real rate of interest. Specifically, we find that the stock returns in these industries have a consistently positive relation with unexpected changes in the real rate of interest for the overall, contraction and expansion sub-periods. This suggests that investments in these industries will tend to increase if real rates of interest unexpectedly rise, thereby offsetting extra costs associated with a rise in the real rate of interest.

Our empirical results support the state-dependent nature of the investor behavior in the interest rate sensitivity analysis. Also, this study may find a herding behavior of investors in some scenarios, because in certain times of market stress, investors disregard their own information, and exhibit herding behavior, which is often extremely optimistic or pessimistic and may lead to an unreasonable reaction to movements in interest rates. Finally, we confirm the null hypothesis that investor behavior may depend on different factors that affect the investment or trading decision. Therefore, aspects such as the sector that traded stock belongs to and the business cycle definitely impact on investment behavior.

## Author contributions

All authors listed, have made substantial, direct and intellectual contribution to the work, and approved it for publication.

### Conflict of interest statement

The authors declare that the research was conducted in the absence of any commercial or financial relationships that could be construed as a potential conflict of interest.
