# Supplementary material for: Interest and Inflation Risk: Investor Behavior
Source: Front Psychol. 2016 Mar 18;7:390. doi: 10.3389/fpsyg.2016.00390 (PMC4796036; doi:10.3389/fpsyg.2016.00390)
Supplement: Supplementary file 1 [file Table1.pdf]

Interest and Inflation Risk: Investor Behavior

María de la O González1, Francisco Jareño1*, Frank S. Skinner2

[1]Department of Economic Analysis and Finance, University of Castilla-La Mancha, Albacete, Spain

[2]Department of Economics and Finance, Brunel University, Uxbridge, Middlesex, United Kingdom

*** Correspondence:** Corresponding Author, Department of Economic Analysis and Finance, University of Castilla-La Mancha, Plaza de la Universidad 1, Albacete, 02071, Spain

francisco.jareno@uclm.es

Keywords: Unexpected inflation, interest rates, Stock return, Business Cycle, Investor behavior.

Abstract

We examine investor behavior under interest and inflation risk in different scenarios. To that end, we analyze the relation between stock returns and unexpected changes in nominal and real interest rates and inflation for the US stock market. This relation is examined in detail by breaking the results down from the US stock market level to sector, sub-sector and to individual industries as the ability of different industries to absorb unexpected changes in interest rates and inflation can vary by industry and by contraction and expansion sub-periods. While most significant relations are conventionally negative, some are consistently positive. This suggests some relevant implications on investor behavior. Thus, investments in industries with this positive relation can form a safe haven from unexpected changes in real and nominal interest rates. Gold has an insignificant beta during recessionary conditions hinting that Gold can be a safe haven during recessions. However, Gold also has a consistent negative relation to unexpected changes in inflation thereby damaging the claim that Gold is a hedge against inflation.

Supplementary material

Table A. The Global Industry Classification Standard (GICS) combined with Bloomberg classification as of April 29, 2010

Panel A Consumer Discretionary, Consumer Staples and Energy

Sector/Sub-sector (Weights)	Industries
**S1 Consumer Discretionary (10.59%)**	
SS1 Automobiles & components	I1 Auto components<br>I2 Automobiles
SS2 Consumer Durables & Apparel	I3 Household Durables<br>I4 Leisure Equipment & Products<br>I5 Textiles, Apparel & Luxury Goods
SS3 Consumer Services	I6 Hotels Restaurants & Leisure<br>I7 Diversified Consumer Services
SS4 Media	I8 Advertising<br>I9 Broadcasting<br>I10 Cable & Satellite<br>I11 Movies & Entertainment<br>I12 Publishing
SS5 Retailing	I13 Distributors<br>I14 Internet & Catalog Retail<br>I15 Multiline Retail<br>I16 Specialty Retail
**S2 Consumer Staples (10.80%)**	
SS1 Food & Staples Retailing	I1 Drug Retail<br>I2 Food Distributors<br>I3 Food Retail<br>I4 Hypermarkets & Super Centers
SS2 Food Beverage & Tobacco	I5 Beverages<br>I6 Food Products<br>I7 Tobacco
SS3 Household & Personal Products	I8 Household Products<br>I9 Personal Products
**S3 Energy (11.50%)**	
SS1 Energy Equipment & Services	I1 Oil & Gas Drilling<br>I2 Oil & Gas Equipment & Services
SS2 Oil, Gas & Consumable Fuels	I3 Integrated Oil & Gas<br>I4 Oil & Gas Exploration & Production<br>I5 Oil & Gas Refining & Marketing<br>I6 Oil & Gas Storage & Transportation<br>I7 Coal & Consumable Fuels

Table A. The Global Industry Classification Standard (GICS) combined with Bloomberg classification as of April 29, 2010 (cont.)

Panel B Financials, Health Care, Industrials and Information Technology

Sector/Sub-sector (Weights)	Industries
**S4 Financials (16.58%)**	
SS1 Banks	I1 Commercial Banks<br>I2 Thrifts & Mortgage Finance
SS2 Diversified Financials	I3 Diversified Financial Services<br>I4 Consumer Finance<br>I5 Capital Markets
SS3 Insurance	I6 Insurance Brokers<br>I7 Life & Health Insurance<br>I8 Multi-line Insurance<br>I9 Property & Casualty Insurance
SS4 Real Estate	I10 Real Estate Investment Trusts (REITs)<br>I11 Real Estate Management & Development
**S5 Health Care (11.50%)**	
SS1 Health Care Equipment & Services	I1 Health Care Equipment & Supplies<br>I2 Health Care Providers & Services
SS2 Pharmaceuticals, Biotechnology & Life Sciences	I3 Biotechnology<br>I4 Pharmaceuticals<br>I5 Life Sciences Tools & Services
**S6 Industrials (10.79%)**	
SS1 Capital Goods	I1 Aerospace & Defense<br>I2 Building Products<br>I3 Construction & Engineering<br>I4 Electrical Equipment<br>I5 Industrial Conglomerates<br>I6 Machinery<br>I7 Trading Companies & Distributors
SS2 Commercial & Professional Services	I8 Commercial Services & Supplies<br>I9 Professional Services
SS3 Transportation	I10 Air Freight & Logistics<br>I11 Airlines<br>I12 Road & Rail
**S7 Information Technology (19.02%)**	
SS1Software & Services	I1 Internet Software & Services<br>I2 IT Services<br>I3 Software
SS2 Technology Hardware & Equipment	I4 Communications Equipment<br>I5 Computers & Peripherals<br>I6 Electronic Equip., Instruments & Components<br>I7 Office Electronics
SS3 Semiconductors & Semiconductor Equipment	I8 Semiconductor Equipment<br>I9 Semiconductors

Table A. The Global Industry Classification Standard (GICS) combined with Bloomberg classification as of April 29, 2010 (cont.)

Panel C Materials, Telecommunications Services and Utilities

Sector/Sub-sector (Weights)	Industries
**S8 Materials (3.44%)**	
SS1 Chemicals	I1 Diversified Chemicals<br>I2 Fertilizers & Agricultural Chemicals<br>I3 Industrial Gases<br>I4 Specialty Chemicals
SS2 Construction Materials	
SS3 Containers & Packaging	I5 Metal & Glass Containers<br>I6 Paper Packaging
SS4 Metals & Mining	I7 Aluminum<br>I8 Diversified Metals & Mining<br>I9 Gold<br>I10 Steel
SS5 Paper & Forest Products	I11 Forest Products<br>I12 Paper Products
**S9 Telecommunications Services (2.71%)**	
SS1 Diversified Telecommunication Services	I1 Integrated Telecommunication Services
SS2 Wireless Telecommunication Services	
**S10 Utilities (3.42%)**	
SS1 Electric Utilities	
SS2 Gas Utilities	
SS3 Multi-Utilities	
SS4 Independent Power Producers & Energy Traders	
